# A Novel GJA8 Mutation (p.V44A) Causing Autosomal Dominant Congenital Cataract

**DOI:** 10.1371/journal.pone.0115406

**Published:** 2014-12-17

**Authors:** Yanan Zhu, Hao Yu, Wei Wang, Xiaohua Gong, Ke Yao

**Affiliations:** 1 Eye center, 2nd Affiliated Hospital of Medical College, Zhejiang University, Hangzhou, China; 2 Key Laboratory of Ophthalmology of Zhejiang Province, Wenzhou, China; 3 Department of Neurobiology, Key Laboratory of Neurobiology of Zhejiang Province, Zhejiang University School of Medicine, Hangzhou, China; 4 School of Optometry and Vision Science Program, University of California, Berkeley, California, United States of America; University of Durham, United Kingdom

## Abstract

**Purpose:**

To examine the mechanism by which a novel connexin 50 (Cx50) mutation, Cx50 V44A, in a Chinese family causes suture-sparing autosomal dominant congenital nuclear cataracts.

**Methods:**

Family history and clinical data were recorded and direct gene sequencing was used to identify the disease-causing mutation. The Cx50 gene was cloned from a human lens cDNA library. Connexin protein distributions were assessed by fluorescence microscopy. Hemichannel functions were analyzed by dye uptake assay. Formation of functional channels was assessed by dye transfer experiments.

**Results:**

Direct sequencing of the candidate *GJA8* gene revealed a novel c.131T>C transition in exon 2, which cosegregated with the disease in the family and resulted in the substitution of a valine residue with alanine at codon 44 (p. V44A) in the extracellular loop 1 of the Cx50 protein. Both Cx50 and Cx50V44A formed functional gap junctions, as shown by the neurobiotin transfer assay. However, unlike wild-type Cx50, Cx50V44A was unable to form open hemichannels in dye uptake experiments.

**Conclusion:**

This work identified a unique congenital cataract in the Chinese population, caused by the novel mutation Cx50V44A, and it showed that the V44A mutation specifically impairs the gating of the hemichannels but not the gap junction channels. The dysfunctional hemichannels resulted in the development of human congenital cataracts.

## Introduction

Congenital cataracts are a worldwide cause of childhood blindness, accounting for more than 1 million cases of pediatric blindness in Asia and approximately 10% of the worldwide cases of childhood blindness [1]. Genetic mutations may be the most common cause of this condition, particularly in cases of bilateral cataracts. Thirty-seven gene mutations are currently known to be associated with congenital or childhood cataracts, approximately one-quarter of which are located in the connexins [2], [3].

Both connexin 46 (Cx46) and connexin 50 (Cx50), which are encoded by the *GJA3* and *GJA8* genes, respectively, have been reported to be associated with autosomal dominant congenital cataracts [4]. Several strains of mice or rats that develop cataracts also harbor mutations of the *GJA3* (*Cx46*) or *GJA8* (*Cx50*) genes [5], [6]. Studies have shown that these transport membrane proteins are important for the correct embryological development of the lens.

Gap junctions, which are channels that connect the cytoplasm of neighboring cells, are well documented to play an important role in intercellular communication in the lens, which is avascular. These junctions are formed by two hemichannels (connexons), which consist of six connexin molecules. Prior to assembly into gap junctional channels, hemichannels transiently reside in the plasma membrane and can open in response to various triggers [7]. Recent studies have proposed a potential role of hemichannels in autocrine/paracrine signaling that is unrelated to their role as precursors in the formation of gap junction channels [8].

In this study, we identified a novel heterozygous mutation, the c.131T>C (p.V44A) mutation, in exon2 of *GJA8* in a Chinese family with suture-sparing nuclear cataracts. In addition, we examined the mechanism of cataract formation in patients carrying the Cx50 V44A mutation. Functional study showed that this mutation caused abnormal alterations in hemichannel function, which may represent a possible mechanism for the formation of cataracts.

## Methods

### Ethics Statement

The study protocol was in accord with the ethical guidelines of the Declaration of Helsinki and was approved by the Ethics Committee of Zhejiang University. Appropriate written informed consent was obtained from all of the adult participants; consent was obtained from adult guardians on behalf of the pediatric participants.

### Clinical Evaluation and Examination

A four-generation Chinese pedigree diagnosed with autosomal dominant congenital cataracts was recruited at the Second Affiliated Hospital of Zhejiang University (Hangzhou, China). Thirty-four individuals (eight affected and 26 unaffected) from the family participated in the study. The affected status was determined by a history of cataract surgery or by ophthalmic examinations.

### Genomic DNA Preparation

We collected blood specimens (5 mL) of all the participants into EDTA tubes. The peripheral blood leukocytes were then used to extract genomic DNA.

### Mutation Screening

We performed the mutation screening using the functional candidate gene analysis approach. Gene-specific PCR primers that flanked each exon and intron-exon junction were designed for genes that were related to nuclear cataract, such as *CRYAA, CRYAB, CRYBA3/A1, CRYBB1, CRYBB2, CRYGC, CRYGD, GJA3, GJA8* and *MIP*. Details of the strategy used for PCR and sequencing are described in our previous study [9]. The results were compared to the NCBI GenBank sequences. Nucleotide numbering reflected the cDNA numbering, with +1 corresponding to the A of the ATG translation initiation codon in the reference sequence. The initiation codon was assigned as codon 1.

### Sequencing and Cloning of Wild-type and Mutant Connexins

The *GJA8* cDNA sequence was cloned from a human lens cDNA library (provided by Graeme Wistow from the NIH as a gift) by PCR using PrimeSTAR HS DNA polymerase (Takara Ltd., Dalian, China) and the following oligo nucleotide primers: sense-primer (5′-GCATTACTCGAGATGGGCGACTGGAGTTTCCTGGG-3′) and antisense-primer (5′-CCATTGAATTCGTACGGTTAGATCGTCTGACCTGGCTCG-3′). After digestion with XhoI and EcoRI (NEB, Ipswich, MA, USA), the PCR product was cloned into the expression vector pEGFP-N1 (Invitrogen, Carlsbad, CA, USA). The resultant construct, Cx50WILD, was confirmed by DNA sequencing.

A Quik-Change Site-Directed Mutagenesis Kit (Stratagene, LaJolla, CA, USA) was used to construct the mutant Cx50V44A plasmid using the following mutagenic primers:5′-CACGGCCGCAGAGTTCGCGTGGGGGGATGAGCAATC-3′ and 5′-GATTGCTCATCCCCCCACGCGAACTCTGCGGCCGTG-3′. The Cx50 mutants were sequenced to verify the target mutation.

### Cell Culture and Transfections

Human epithelial carcinoma cells (HeLa, ATCC) were grown in DMEM (Gibco, Carlsbad, CA, USA) with 10% FBS. Transfection was performed on HeLa cells using the transfection reagent Lipofectamine 2000 (Invitrogen), according to the manufacturer's instructions. HeLa cell monoclones that stably expressed human wild-type Cx50, mutant Cx50V44A or the EGFP control were isolated based on their resistance to geneticin (G418) (1 mg/mL). To maintain stability of expression of the target proteins, G418 (0.5 mg/mL) was used during cell culturing.

### Identification the Connexin43 Status of Transfected HeLa Cells

Cx50WILD-transfected, Cx50V44A-transfected, EGFP-transfected HeLa cells and human retinal epithelial cells (ARPE-19, ATCC) were seeded in 100-mm dishes for 24 h. ARPE-19, which has been reported to express Cx43 [10], served as a control. The cells were collected in ice-cold PBS and were then centrifuged at 1000 rpm at 4°C for 5 min. After removing the supernatant, total protein was extracted from the cells with a lysis buffer (Sangon Biotech, Shanghai, China). After incubation on ice for 30 min, the extracts were centrifuged at 14,000 rpm at 4°C for 15 min. Then, the supernatant of each sample was transferred to a fresh tube and boiled with a protein-loading buffer (Sangon Biotech). The proteins were separated by 10% SDS-polyacrylamide gel electrophoresis, transferred to polyvinylidene difluoride membranes and blotted with anti-Cx43 rabbit polyclonal antibody (1∶200 dilution, Santa Cruz) and anti-GAPDH rabbit monoclonal antibody (1∶5000 dilution, Cell Signaling Technology, USA). After incubation in fluorescent secondary antibodies (1∶5000 dilution, Cell Signaling), the blots were analyzed using the Bio-Rad ChemiDoc MP imaging system. The target protein expression levels were normalized relative to GAPDH expression.

### Identification of Cx50 Status of Transfected Cells

The expression levels of Cx50WILD and Cx50V44A in stably transfected HeLa cells were verified by Western blot, and the EGFP-transfected cells served as controls. The target protein was blotted with anti-Cx50 rabbit polyclonal antibody (1∶200 dilution, Santa Cruz) and with fluorescent secondary antibodies, and its expression level was normalized relative to GAPDH expression.

### Fluorescence Imaging

Stably transfected cells that expressed the GFP-tagged connexins were plated on four-well chamber slides (Lab Tek; Nalge Nunc International, Naperville, IL, USA). Fluorescence images were obtained when the cultured cells reached 80% to 90% confluence. Cx50 protein distribution and gap junction formation were monitored based on tagged GFP signaling in these cells using a confocal laser scanning microscope (×40, Zeiss LSM 510, Zurich, Switzerland).

### Dye Uptake Experiments

For hemichannel function analysis, three groups of cells (Cx50WILD, Cx50V44A, and control) were placed in a 35-mm collagen-coated glass-bottom culture dish (50% confluence). The cells were washed twice with Ca^2+^-free HBSS (Gibco). Dye uptake assays were performed by incubating each group of cells in Ca^2+^-free HBSS (Gibco) and in HBSS containing 1.2 mmol/L Ca^2+^ or a specific hemichannel blocker, flufenamic acid (FFA, 300 µmol/L, Sigma, Saint Louis, MO, USA) [11]. All of the solutions were supplemented with 0.1% propidium iodide (PI, Sigma) or 0.1% 4′,6-diamidino-2-phenylindole (DAPI, Sigma). After 30 min, the solutions were removed; the cells were then washed with PBS, twice, and finally incubated in normal culture solutions for counting. Excluding the dead cells that either lost their normal shape or became unattached, the total numbers of live cells and dye-loading live cells were counted using fluorescence microscopy (×40). Thirty fields were applied each time, and the dye uptake experiments were repeated three times. The results are shown as percentages of loading cells and are expressed as the means ± the standard deviations (SDs). Statistical analysis was performed using Student's t-test.

### *In situ* Dye Transfer Experiments

The cells were cultured with normal culture medium on glass coverslips until 80%–90% confluence was reached. Then, the glass coverslips were transferred to a bath solution containing 140 mM NaCl, 1 mM MgCl_2_, 5.4 mM KCl, 1.2 mM CaCl_2_, and 10 mM HEPES (pH 7.2). The cells were impaled with a micropipette filled with a patch pipette solution and 4% neurobiotin dye (Mr = 322.8, charge  = +1; Vector Laboratories, Burlingame, CA, USA) or 5% lucifer yellow dye (Mr = 457, charge  = −2; Invitrogen). The patch pipette solution contained 140 mM KCl, 1 mM MgCl_2_, 5 mM NaCl, and 10 mMHEPES (pH 7.4). The dye solution was microinjected for 3 min with a Picospritzer (model PLI-188; Nikon, Tokyo, Japan).

After neurobiotin dye injection, the cells were fixed, permeabilized, and stained with Cy3-streptavidin conjugate (Sigma) for tracer detection. However, after lucifer yellow dye injection, the cells required only fixation. Both types of cells were then photographed with fluorescence microscopy. The adjacent cells stained by the dye were counted to determine the extent of the intercellular tracer transfer. Three independent stable-cell clones were all used to perform the dye transfer assay. The number of coupled cells was expressed as the means ±SDs. Statistical analysis was performed with Student's t-test.

## Results

### Clinical Evaluation

**Figure 1:**
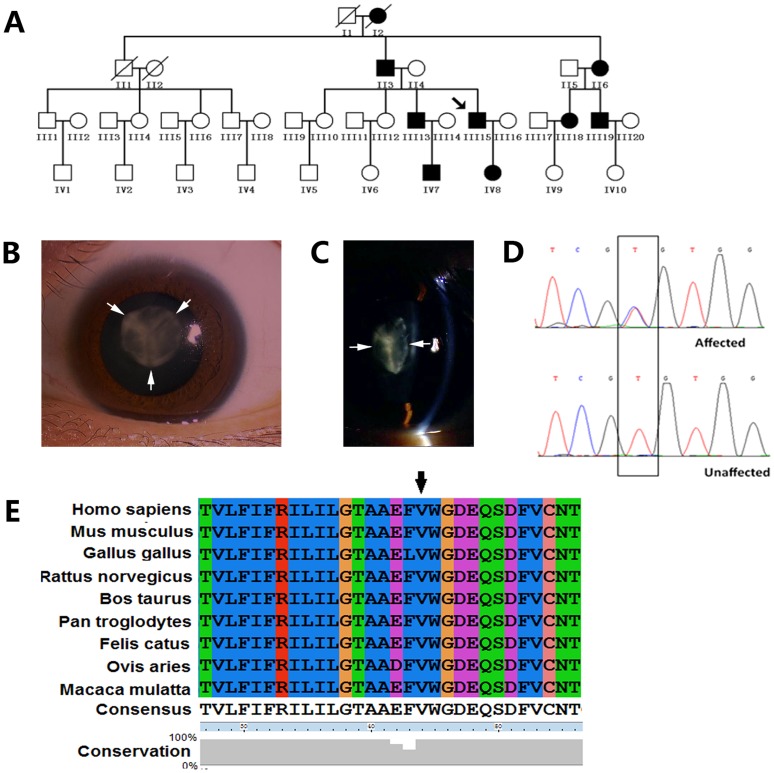
Family data collection, cataract phenotype, and mutation sequencing of Cx50. A: Pedigree of the proband. The black symbols indicate the affected individuals. The arrow indicates the proband. All the living members shown in this figure participated in this project. B: Diffuse illumination of the left eye of the proband (the arrows show transparent anterior suture). C: Slit lamp photograph of the lens of the proband showing the nuclear opacities; anterior and posterior sutures are not involved (arrowheads). D: Partial DNA sequence of *GJA8* from one normal individual and one affected individual. The black box shows a heterozygous mutation (c.131T>C) in exon 2. E: Conservation analysis of p.V44A of the Cx50 gene.

We identified a four-generation Chinese family with autosomal dominant congenital cataracts. The majority of the patients realized their visual impairment before the age of 10, particularly during the daytime. The visual acuity decreased gradually, and cataract surgery was eventually performed at approximately 40 years of age (Fig. 1A). The affected individuals all presented with symmetrical, homogeneous nuclear opacification in both eyes. The anterior and posterior Y-sutures were not involved (Fig. 1B, C). The patients had best-corrected visual acuity (BCVA) values that ranged from finger counting to 0.2 prior to surgery, and they achieved BCVA values ranging from 0.5 to 0.8 after surgery. In their family, the unaffected members had good BCVA values ranging from 0.6 to 1.0. There was no family history of other ocular or systemic abnormalities.

### Genetic Analysis

Using gene sequencing, we identified a novel heterozygous c.131T>C mutation in the *GJA8* gene (RefSeq NM_005267.4). This variation occurred in all of the affected individuals (Fig. 1D), but it was not observed in the unaffected family members or in the 100 unrelated healthy individuals who served as controls. This mutation resulted in a substitution of a valine residue for alanine at codon 44 of connexin 50 (p.V44A), which is located in the first extracellular loop of this protein [12]. Multiple sequence alignment indicates that the mutation is located within a highly conserved region (Fig. 1E).

### Function Analysis

**Figure 2:**
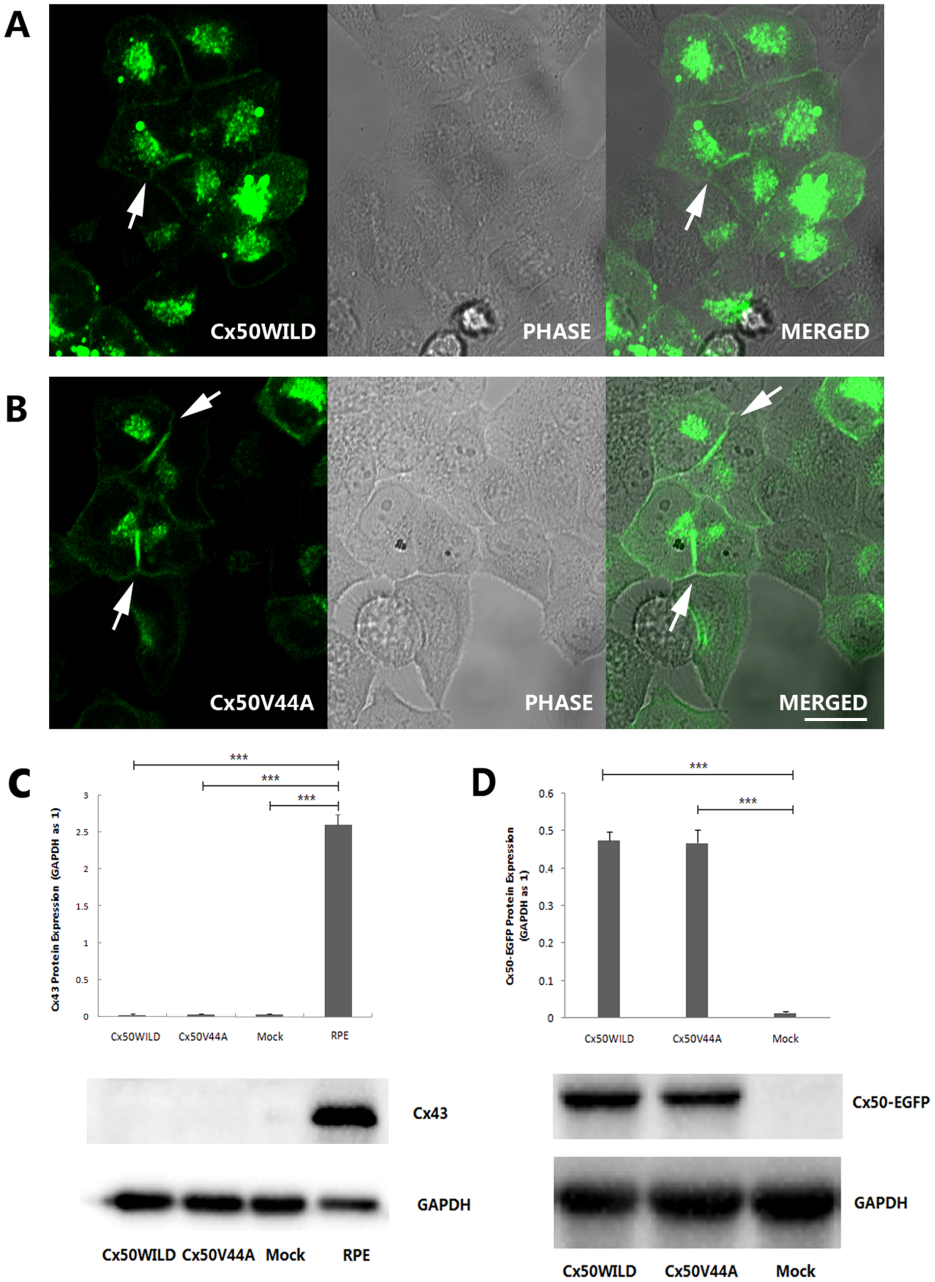
Subcellular localization of Cx50WILD and Cx50V44A in stably transfected HeLa cells. Both Cx50WILD (A) and Cx50V44A (B) were observed at appositional membranes and perinuclear cytoplasmic locations (the arrows show the gap junction plaques). Scale bar: A-B 20 µm. C: Endogenous expression of Cx43 in transfected HeLa cells. The transfected cells did not express Cx43 protein, but the ARPE-19 cells (RPE) did. (*P*<0.001, marked with ***) D: The stable transfected cells expressed similar protein levels for Cx50WILD or Cx50V44A.

HeLa cells from ATCC have been reported to express Cx43 in early generations, but in our experiment, the transfected HeLa cells that were used did not express the Cx43 protein (Fig. 2C). Therefore, these transfected HeLa cells were good models because of their connexin deficiency [13]. GFP-tagged versions of Cx50WILD or Cx50V44A were expressed in HeLa cells, and similar levels of the expression of wild-type protein or mutant protein of approximately 98 kDa were detected by Western blot, whereas no expression of Cx50 was detected in the control cells (Fig. 2D). Cellular distributions of the GFP-tagged Cx50WILD and Cx50V44A proteins were determined based on their GFP fluorescence. As expected, the wild-type Cx50 proteins formed gap junctional plaques on appositional membranes and were present in the cytoplasmic and perinuclear regions (Fig. 2A). The Cx50V44A mutants also formed typical gap junctions and were present in the cytoplasmic and perinuclear regions, similar to wild-type Cx50 (Fig. 2B). The results indicate that the V44A mutation did not affect Cx50 protein trafficking and gap junction formation.

**Figure 3:**
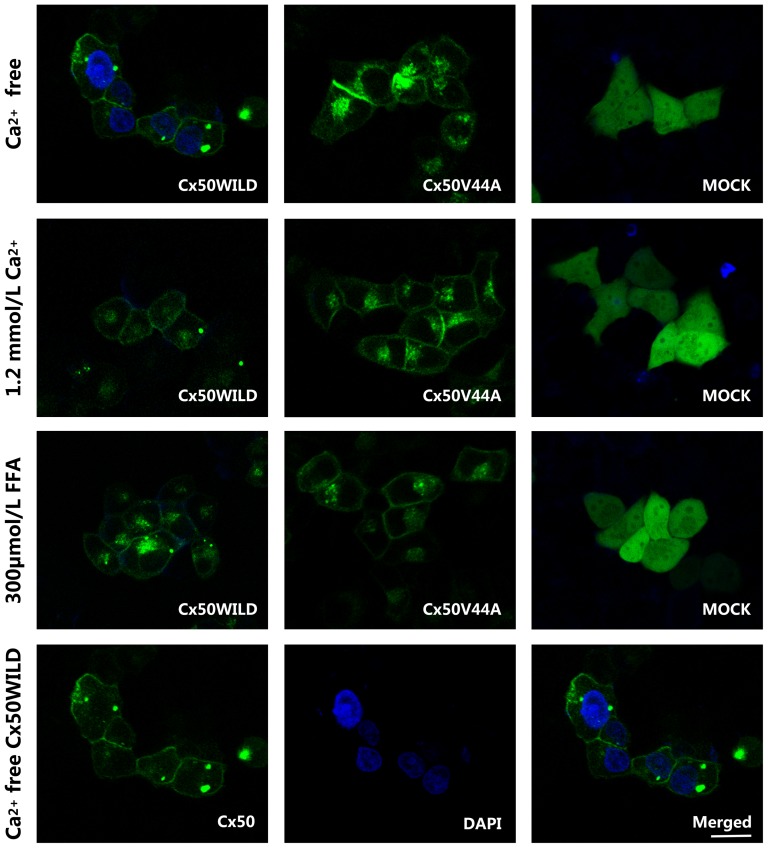
DAPI dye uptake in HeLa cells stably transfected with Cx50WILD and Cx50V44A. DAPI was abosorbed by most of the Cx50WILD-transfected cells, but not by the Cx50V44A- and pEGFP-N1-transfected cells (Mock), after hemichannel opening. DAPI loading in Ca^2+^-free HBSS was blocked by 1.2 mM Ca^2+^ and 300 µM FFA. The three pictures in the last line show the Cx50 protein, DAPI-stained nuclei and merged look of the wild-type cells in Ca^2+^-free HBSS. Scale bar: 20 µm.

**Figure 4:**
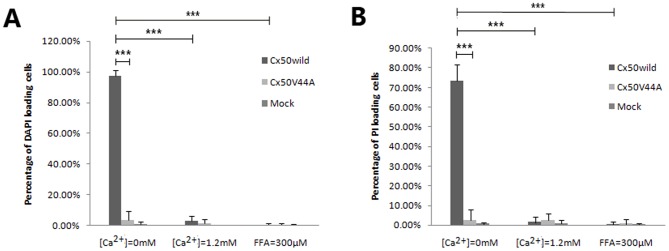
PI dye uptake in HeLa cells stably transfected with Cx50WILD and Cx50V44A. The PI dye uptake assay revealed similar results as the DAPI dye uptake assay. The three pictures in the last line show the Cx50 protein, PI-stained nuclei (nucleoli)/cytoplasm, and merged look of the wild-type cells in Ca^2+^-free HBSS. Scale bar: 20 µm.

**Figure 5:**
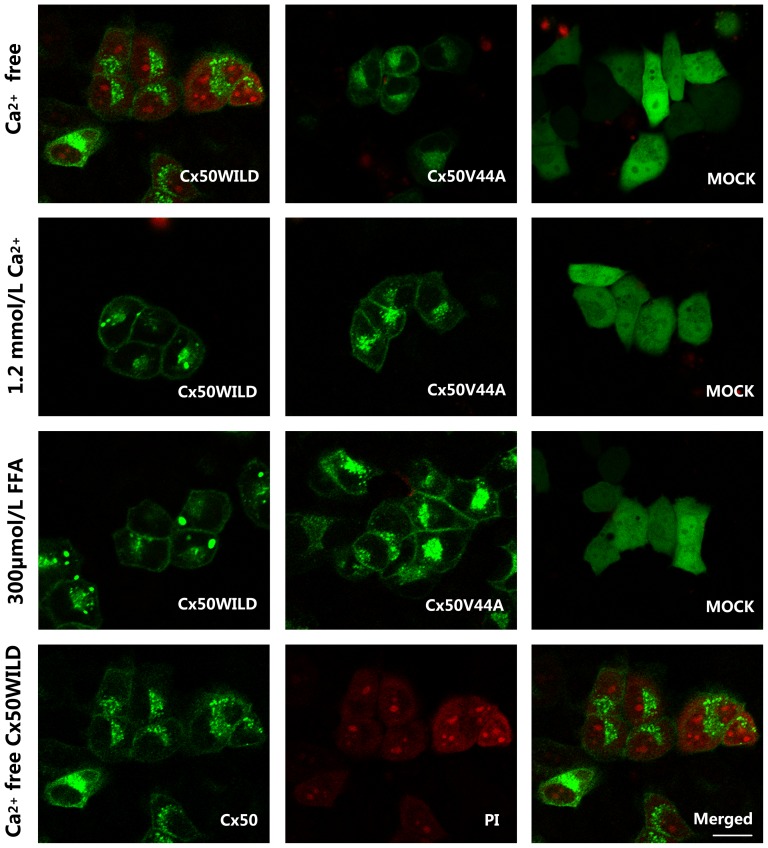
Statistical analysis of DAPI (A) and PI (B) loading in different solutions. Data are presented as mean±SDs. There was a significant difference in the percentage of dye-stained cells between the Cx50WILD and Cx50V44A groups in the Ca^2+^-free environment (*P*<0.001, marked with ***); there was also a significant difference between the Ca^2+^-free Cx50WILD and the 1.2 mM Ca^2+^/300 µM FFA Cx50WILD group (*P*<0.001, marked with ***).

We then examined hemichannel function. Dye (such as DAPI or PI) uptake assays revealed that wild-type Cx50 hemichannels opened in Ca^2+^-free HBSS medium after 30 min of treatment and closed after incubation in HBSS containing 1.2 mmol/L Ca^2+^ or 300 µmol/L FFA for the same period of time. As expected, the DAPI uptake assay confirmed that almost all of the Cx50WILD cells (97.5±3.63%) contained high-intensity blue fluorescence in their nuclei, whereas a low percentage of DAPI-stained cells was observed in the Cx50V44A cells (3.72±5.91%) and the control cells (1.10±1.48%). After incubation in HBSS containing 1.2 mmol/L Ca^2+^ for the same duration, very few Cx50WILD cells (3.00±3.07%) contained fluorescent dye, which was similar to the Cx50V44A cells (1.34±3.04%) and the control cells (0.17±0.52%) (Figs. 3 and 4). The PI uptake assay revealed similar results for the Cx50, Cx50V44A and control cells. Most of the Cx50WILD cells(73.57±8.21%) showed high-intensity red fluorescence in their nuclei, particularly in the nucleoli, and low-intensity red fluorescence in the cytoplasm, whereas a very low percentage of the Cx50V44A (2.59±5.33%) and control cells (0.67±0.96%) were positive for PI staining. After incubation in HBSS containing 1.2 mmol/L Ca^2+^ for the same amount of time, very few of the Cx50WILD cells (1.46±2.86%), Cx50V44A cells (2.44±3.50%), and control cells (0.84±1.59%) absorbed PI (Figs. 4 and 5). Hemichannel function was assured using FFA. Dye uptake was completely blocked by 300 µmol/L FFA. In the FFA-treated cells, only 0.32±1.39% of the Cx50WILD cells, 0.67±2.41% of the Cx50V44A cells and 0.32±0.74% of the control cells absorbed the PI dye, while 0.41±1.34% of Cx50WILD cells, 0.56±0.95% of the Cx50V44A cells and 0.41±0.80% of the control cells absorbed the DAPI dye. (Figs. 3 and 5) Thus, these results suggest that the Cx50V44A mutant proteins were unable to form calcium-sensitive hemichannels, such as those formed by wild-type Cx50.

**Figure 6:**
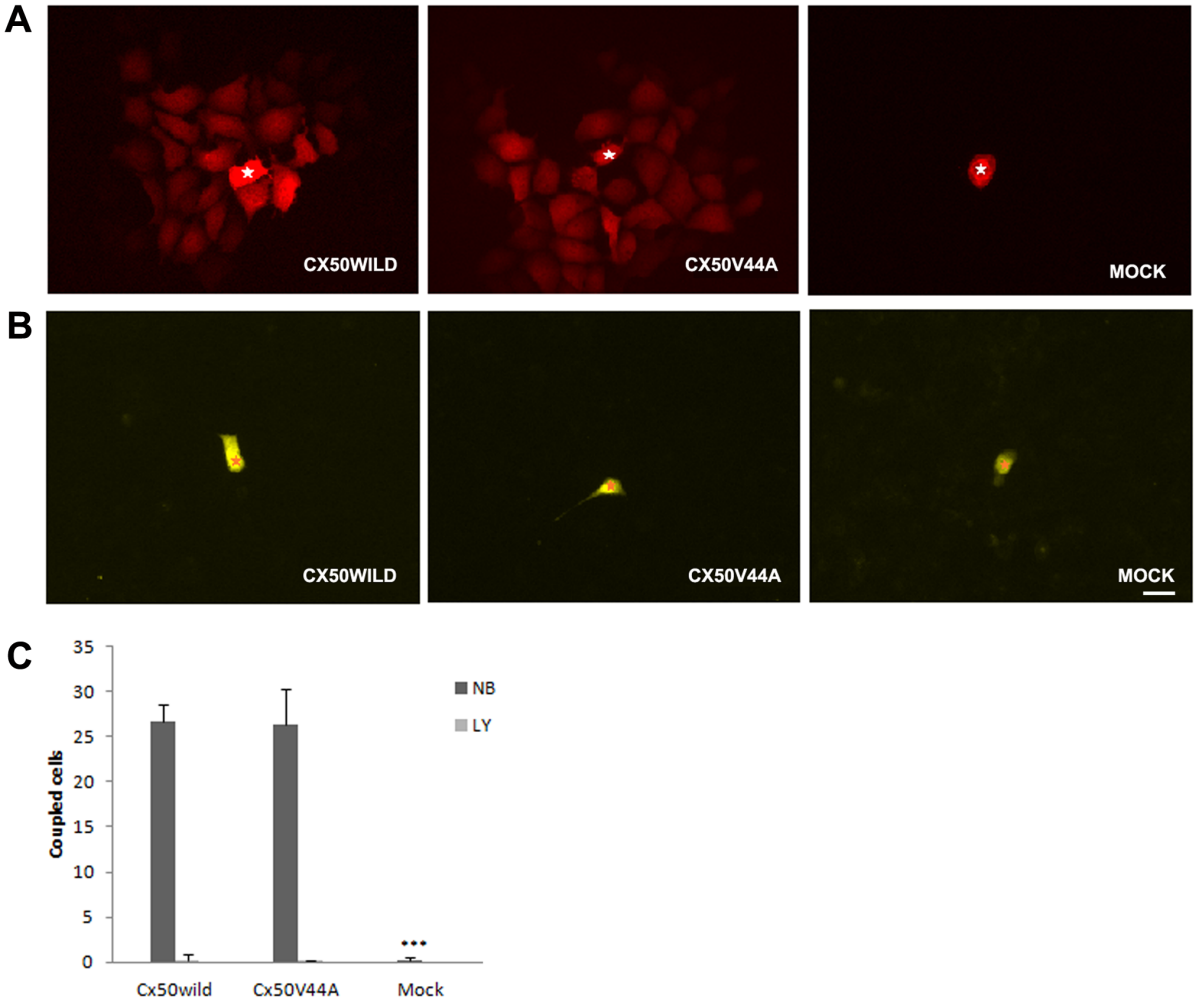
Dye transfer assay in HeLa cells stably transfected with Cx50WILD and Cx50V44A. A: Cx50WILD- and Cx50V44A-transfected cells displayed extensive transfer of neurobiotin, whereas the pEGFP-N1-transfected cells did not allow transfer of this tracer. The white stars show the injected cells. (B) Neither cell line showed significant intercellular transfer of Lucifer yellow. The orange stars show the injected cells. Scale bar: A-B 20 µm. (C) The number of three groups of cells absorbing neurobiotin (NB) and lucifer yellow (LY) from the injected cell. Data are presented as mean±SDs; n = 20; the neurobiotin transfer assay showed significant differences in the percentage of coupled cells between the control group and the Cx50WILD/Cx50V44A groups (*P*<0.001, marked with ***).

We further tested the ability of Cx50V44A to form functional gap junction channels by using dye microinjection of gap junction tracers in individual cells. We quantified the intercellular gap junction communication by counting the number of tracer-containing neighboring cells. Neurobiotin transfer was detected in the Cx50WILD cells (26.75±1.89coupled cells; the number of microinjected cells (n) = 20). Surprisingly, the neurobiotin transfer data were similar in the Cx50V44A cells (26.33±3.98 coupled cells; n = 20). The control cells showed almost no transfer of neurobiotin (0.15±0.37 coupled cells, n = 20) (Fig. 6A, C). As expected, both the Cx50 and Cx50V44A cells did not transfer Lucifer yellow dye (Fig. 6B, C). These results indicated that the V44A mutation did not affect the intercellular dye transfer of the Cx50 gap junction channels.

## Discussion

In this study, we described the suture-sparing nuclear cataract phenotype in a Chinese population and identified a novel T→C mutation in the second exon of the *GJA8* gene that was associated with this phenotype. Interestingly, this special cataract phenotype was also previously reported to be related to the *CRYBA3/A1* gene [14], indicating that similar types of cataracts could be caused by different gene mutations.

Our data indicated that the p.V44Amutation in the *GJA8* gene was the causative mutation in this ADCC family, rather than a rare polymorphism in the normal population. First, the mutation cosegregated with the disease phenotype in the family and it was absent in the 100 unaffected, unrelated individuals who were screened as the control. Second, the valine residue at this site was highly conserved because it was present in the sequences of all of the Cx50 orthologs that were deposited in the database. Third, the mutant Cx50 displayed a dysfunctional hemichannel.

The *GJA8* gene, which encodes Cx50, has been heavily investigated in congenital cataracts. To date, 22 Cx50 mutations have been identified in human congenital cataract pedigrees [4], [15], [16]. Cx50, like other connexins, includes an amino terminus (NT), a carboxyl terminus (CT), and four transmembrane domains (TMs) that are linked by a single cytoplasmic loop (CL) and by two extracellular domains (E) [17]. The V44A mutation was located in the extracellular loop 1 near the TM1/E1 border. The importance of this region was further emphasized by its identification at position 42–51 of Cx50 (V44E, W45S, G46V, D47A, D47Y, D47N, D47H, E48K, S50P) [5], [16], [18]–[24]. Among the Cx50 human mutations previously described, Cx50E48K and Cx50D47N showed a decreased ability to form functional channels [23], [24], while Cx50G46V formed enhanced functional channels [20]. Recently, Rubinos reported that Cx50V44E, which was at the same location as the mutation described in the present study, failed to form functional homotypic or heterotypic gap junctions and strongly inhibited WT connexin function [25].

We examined the effects of the novel cataract-associated mutation Cx50V44A. Our results showed that Cx50V44A–mutant proteins were able to traffic and oligomerize into functional gap junctions in the plasma membrane in transfected HeLa cells. However, the Cx50V44A mutants failed to form appropriate functional hemichannels. The electrophysiological data collected *in vitro* demonstrated that hemichannels opened in reduced external calcium [26], [27] and closed in a solution containing 1.2 mmol/L Ca^2+^ [27]. Here, we showed that the opened Cx50V44A hemichannel could not absorb either DAPI dye (MW = 350 Da, charge, +2) or PI dye (MW = 668 Da, charge, +2), whereas Cx50WILD could. These results suggested that, in a Ca^2+^-free environment, the pores of the mutant hemichannels could be closed or be smaller in diameter than the size of the DAPI dye.

The dysfunctional hemichannel caused by Cx50V44A plays an important role in cataract formation. Although the concentration of calcium in the human aqueous humor is ∼1.3 mM [28], calcium homeostasis in the central mature fibers of the lens appears to be more complex. The interfiber calcium concentration in the lens remains unknown. Gao reported that the intracellular calcium concentration in mouse lens varied from 700 nM in the center to 300 nM at the surface [29]. In the physiological environment, perhaps, extracellular calcium concentrations in human lenses are somewhere between ∼1.3 mM calcium in the aqueous humor and intracellular calcium concentration (300–700 nM) in the lens fiber cells. Thus, wild-type hemichannels are probably regulated by the dynamic ranges of extracellular calcium in the lens. However, the Cx50V44A mutated hemichannels are unable to be gated by extracellular calcium levels in the lens. Future studies will be needed to determine the extracellular calcium concentrations in different parts (superficial region, deep cortex and nucleus) of the lens.

The valine at position 44 is known to form part of a hydrophobic core that participates in intraprotomer interactions, which are believed to be important in the stabilization of the connexin structure [30]. Rubinos suggested that substitution of the valine with a charged residue (glutamicacid) could interfere with connexin oligomerization, resulting in a loss of function [25]. However, unlike Cx50V44E, the ability of our mutation to transfer neurobiotin indicated that the Cx50V44A mutation succeeded in achieving functional gap junctions [31]. Moreover, our mutation caused a suture-sparing nuclear cataract, which was much milder than the total cataracts that are caused by Cx50V44E. Alanine, which is an uncharged residue like valine, could not influence connexin oligomerization or twist the orientation of docking, so the mutant connexin remained capable of forming gap junctions.

Hemichannels were once believed to function only when two such channels formed an intercellular channel (the gap junction) between closely apposed cells, containing an aqueous pore exclusive of the extracellular space [32], [33]. However, not all hemichannels are destined to become components of a gap junction channel, and research has suggested that hemichannels play an important role in cellular processes, such as volume regulation [34], the influx/efflux of metabolically relevant solutes such as ATP [35], and cell death [36], [37]. Furthermore, a recent review also suggested that hemichannels are a significant source of autocrine and paracrine messengers [38]. Dysfunctional connexin hemichannels have been reported to be related to cataracts [39]. Cx50G46V forms normal gap junctions, but it increased hemichannel activity [20]. Cx46G2N reduces hemichannel permeability, but it still forms gap junction plaques [11]. Moreover, Cx46G143R increases hemichannel function and causes apoptosis, even when the transfected cells are cultured at low density with minimal gap junctions [40]. Because the V44A mutations cause a loss and/or a reduction in hemichannel activity, they could also cause a metabolic imbalance within the lens, such as a disruption of the lens circulation current that could trigger the development of cataracts.

In summary, we identified a novel heterozygous c.131T>C (p.V44A) mutation in *GJA8* in a family of Chinese origin with congenital cataracts. Our study indicated that residue V44 of Cx50 might be vital in hemichannel formation; however, this mutation did not impair the function of the gap junction channels. Abnormal activity of the hemichannels could disrupt cellular homeostasis, subsequently causing cataracts.

## References

[1] Francis PJ, Berry V, Bhattacharya SS, Moore AT (2000) The genetics of childhood cataract. J Med Genet 37:481–488. 10882749 10.1136/jmg.37.7.481 PMC1734631

[2] Hejtmancik JF (2008) Congenital cataracts and their molecular genetics. Semin Cell Dev Biol 19:134–149. 18035564 10.1016/j.semcdb.2007.10.003 PMC2288487

[3] Reis LM, Tyler RC, Muheisen S, Raggio V, Salviati L, et al (2013) Whole exome sequencing in dominant cataract identifies a new causative factor, CRYBA2, and a variety of novel alleles in known genes. Hum Genet 132(7):761–770. 23508780 10.1007/s00439-013-1289-0 PMC3683360

[4] Jiang JX (2010) Gap Junctions or Hemichannel-Dependent and Independent Roles of Connexins in Cataractogenesis and Lens Development. Curr Mol Med 10(9):851–863. 21091421 10.2174/156652410793937750 PMC6263138

[5] Gong X, Cheng C, Xia CH (2007) Connexins in lens development and cataractogenesis. J Membr Biol 218(1–3):9–12. 17578632 10.1007/s00232-007-9033-0

[6] DeRosa AM, Meşe G, Li L Sellitto C, Brink PR, et al (2009) The cataract causing Cx50-S50P mutant inhibits Cx43 and intercellular communication in the lens epithelium. Exp Cell Res 315(6):1063–1075. 19331825 10.1016/j.yexcr.2009.01.017 PMC2670955

[7] Evans WH, De Vuyst E, Leybaert L (2006) The gap junction cellular internet: connexin hemichannels enter the signalling limelight. Biochem J 397:1–14. 16761954 10.1042/BJ20060175 PMC1479757

[8] Valiunas V (2013) Cyclic nucleotide permeability through unopposed connexin hemichannels. Front Pharmacol 4:75. 23760880 10.3389/fphar.2013.00075 PMC3674318

[9] Zhu Y, Shentu X, Wang W, Li J, Jin C, et al (2010) A Chinese family with progressive childhood cataracts and IVS3+1G>A CRYBA3/A1 mutations. Mol Vis 16:2347–53. 21139983 PMC2994768

[10] Pocrnich CE, Shao Q, Liu H, Feng MM, Harasym S, et al (2012) The effect of connexin43 on the level of vascular endothelial growth factor in human retinal pigment epithelial cells. Graefes Arch Clin Exp Ophthalmol 250(4):515–22. 22138732 10.1007/s00417-011-1871-x

[11] Yao K, Wang W, Zhu Y, Jin C, Shentu X, et al (2011) A novel GJA3 mutation associated with congenital nuclear pulverulent and posterior polar cataract in a Chinese family. Hum Mutat 32(12):1367–70. 21681855 10.1002/humu.21552

[12] Xia CH, Liu H, Cheung D, Cheng C, Wang E, et al (2006) Diverse gap junctions modulate distinct mechanisms for fiber cell formation during lens development and cataractogenesis. Development 133(10):2033–2040. 16611690 10.1242/dev.02361

[13] Hunter AW, Jourdan J, Gourdie RG (2003) Fusion of GFP to the carboxyl terminus of connexin43 increases gap junction size in HeLa cells. Cell Commun Adhes 10(4–6):211–4. 14681018 10.1080/cac.10.4-6.211.214

[14] Ferrini W, Schorderet DF, Othenin-Girard P, Uffer S, Héon E, et al (2004) CRYBA3/A1 gene mutation associated with suture-sparing autosomal dominant congenital nuclear cataract: a novel phenotype. Invest Ophthalmol Vis Sci 45(5):1436–1441. 15111599 10.1167/iovs.03-0760

[15] Santana A, Waiswo M (2011) The genetic and molecular basis of congenital cataract. Arq Bras Oftalmol 74(2):136–142. 21779674 10.1590/s0004-27492011000200016

[16] Li J, Wang Q, Fu Q, Zhu Y, Zhai Y, et al (2013) A novel connexin 50 gene (gap junction protein, alpha 8) mutation associated with congenital nuclear and zonular pulverulent cataract. Mol Vis 19:767–774. 23592913 PMC3626375

[17] Harris AL (2001) Emerging issues of connexin channels: biophysics fills the gap. Q Rev Biophys 34:325–472. 11838236 10.1017/s0033583501003705

[18] Devi RR, Vijayalakshmi P (2006) Novel mutations in GJA8 associated with autosomal dominant congenital cataract and microcornea. Mol Vis 12:190–195. 16604058

[19] Vanita V, Singh JR, Singh D, Varon R, Sperling K (2008) A novel mutation in GJA8 associated with jellyfish-like cataract in a family of Indian origin. Mol Vis 14:323–326. 18334946 PMC2255026

[20] Minogue PJ, Tong JJ, Arora A, Russell-Eggitt I, Hunt DM, et al (2009) A mutant connexin50 with enhanced hemichannel function leads to cell death. Invest Ophthalmol Vis Sci 50(12):5837–5845. 19684000 10.1167/iovs.09-3759 PMC2788668

[21] Xu X, Ebihara L (1999) Characterization of a mouse Cx50 mutation associated with the No2 mouse cataract. Invest Ophthalmol Vis Sci 40(8):1844–1850. 10393059

[22] Lin Y, Liu NN, Lei CT, Fan YC, Liu XQ, et al (2008) A novel GJA8 mutation in a Chinese family with autosomal dominant congenital cataract. Zhonghua Yi Xue Yi Chuan Xue Za Zhi 25(1):59–62. 18247306

[23] Arora A, Minogue PJ, Liu X, Addison PK, Russel-Eggitt I, et al (2008) A novel connexin50 mutation associated with congenital nuclear pulverulent cataracts. J Med Genet 45(3):155–160. 18006672 10.1136/jmg.2007.051029 PMC2756454

[24] Banks EA, Toloue MM, Shi Q, Zhou ZJ, Liu J, et al (2009) Connexin mutation that causes dominant congenital cataracts inhibits gap junctions, but not hemichannels, in a dominant negative manner. J Cell Sci 122(Pt 3):378–388. 19126675 10.1242/jcs.034124 PMC2650834

[25] Rubinos C, Villone K, Mhaske PV, White TW, Srinivas M (2014) Functional effects of Cx50 mutations associated with congenital cataracts. Am J Physiol Cell Physiol 306(3):C212–20. 24005045 10.1152/ajpcell.00098.2013 PMC3920000

[26] Beahm DL, Hall JE (2002) Hemichannel and junctional properties of connexin 50. Biophys J 82(4):2016–2031. 11916859 10.1016/S0006-3495(02)75550-1 PMC1301997

[27] Zhang X, Zou T, Liu Y, Qi Y (2006) The gating effect of calmodulin and calcium on the connexin50 hemichannel. Biol Chem 387(5):595–601. 16740131 10.1515/BC.2006.076

[28] Duncan G, Jacob TJC (1984) Calcium and the physiology of cataract. Ciba Found Symp 106:132–152. 6096095 10.1002/9780470720875.ch8

[29] Gao J, Sun X, Martinez-Wittinghan FJ, Gong X, White TW, et al (2004) Connections between connexins, calcium, and cataracts in the lens. J Gen Physiol 124(4):289–300. 15452195 10.1085/jgp.200409121 PMC2233908

[30] Maeda S, Nakagawa S, Suga M, Yamashita E, Oshima A, et al (2009) Structure of the connexin 26 gap junction channel at 3.5 A resolution. Nature 458:597–602. 19340074 10.1038/nature07869

[31] Thomas BC, Minogue PJ, Valiunas V, Kanaporis G, Brink PR, et al (2008) Cataracts Are Caused by Alterations of a Critical N-Terminal Positive Charge in Connexin50. Invest Ophthalmol Vis Sci 49(6):2549–2556. 18326694 10.1167/iovs.07-1658 PMC2694449

[32] Maurer P, Weingart R (1987) Cell pairs isolated from adult guinea pig and rat hearts: effects of [Ca^2+^]_i_ on nexal membrane resistance. Pflugers Arch 409:394–402. 3627957 10.1007/BF00583793

[33] Bukauskas FF, Jordan K, Bukauskiene A, Bennett MV, Lampe PD, et al (2000) Clustering of connexin43-enhanced green fluorescent protein gap junction channels and functional coupling in living cells. Proc Natl Acad Sci USA 97:2556–2561. 10706639 10.1073/pnas.050588497 PMC15967

[34] Quist AP, Rhee SK, Lin H, LaI R (2000) Physiological role of gap-junctional hemichannels. Extracellular calcium-dependent is osmotic volume regulation. J CellBiol 148:1063–1074. 10.1083/jcb.148.5.1063 PMC2174555 10704454

[35] Bruzzone S, Franco L, Guida L, Zocchi E, Contini P, et al (2001) A self-restricted CD38-connexin43 cross-talk affects NAD^+^ and cyclic ADP-ribose metabolism and regulates intracellular calcium in 3T3 fibroblasts. J Biol Chem 276:48300–48308. 11602597 10.1074/jbc.M107308200

[36] Plotkin LI, Manolagas SC, Bellido T (2002) Transduction of cell survival signals by connexin-43 hemichannels. J Biol Chem 277:8648–8657. 11741942 10.1074/jbc.M108625200

[37] Kalvelyte A, Imbrasaite A, Bukauskiene A, Verselis VK, Bukauskas FF (2003) Connexins and apoptotic transformation. Biochem Pharmacol 66:1661–1672. 14555247 10.1016/s0006-2952(03)00540-9 PMC3689318

[38] Wang N, DeBock M, Decrock E, Bol M, Gadicherla A, et al (2013) Paracrine signaling through plasma membrane hemichannels. Biochim Biophys Acta 1828:35–50. 22796188 10.1016/j.bbamem.2012.07.002 PMC3666170

[39] Beyer EC, Berthoud VM (2014) Connexin_hemichannels in the lens. Front Physiol 5:20. 24575044 10.3389/fphys.2014.00020 PMC3920103

[40] Ren Q, Riquelme MA, Xu J, Yan X, Nicholson BJ, et al (2013) Cataract-causing mutation of human connexin 46 impairs gap junction, but increases hemichannel function and cell death. PLoS One 8(9):e74732. 24019978 10.1371/journal.pone.0074732 PMC3760834

