# Correction: A Novel GJA8 Mutation (p.V44A) Causing Autosomal Dominant Congenital Cataract

**DOI:** 10.1371/journal.pone.0125949

**Published:** 2015-05-13

**Authors:** Yanan Zhu, Hao Yu, Wei Wang, Xiaohua Gong, Ke Yao

The legends for Fig 4 and Fig 5 are incorrectly switched. Please see the complete, corrected Fig 4 here.

**Fig 4:**
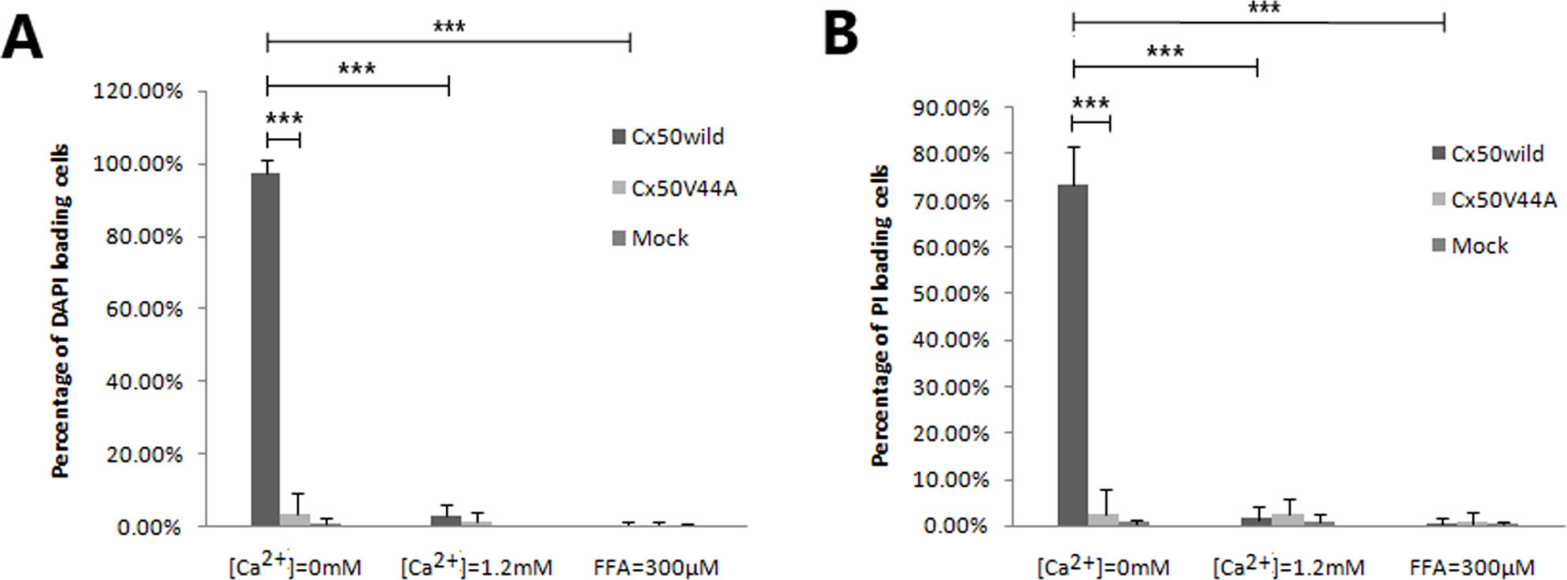
Statistical analysis of DAPI (A) and PI (B) loading in different solutions. Data are presented as mean±SDs. There was a significant difference in the percentage of dye-stained cells between the Cx50WILD and Cx50V44A groups in the Ca^2+^-free environment (*P*<0.001, marked with ***); there was also a significant difference between the Ca^2+^-free Cx50WILD and the 1.2 mM Ca^2+^/300 μM FFA Cx50WILD group (*P*<0.001, marked with ***).

Please see the complete, corrected Fig 5 here.

**Fig 5:**
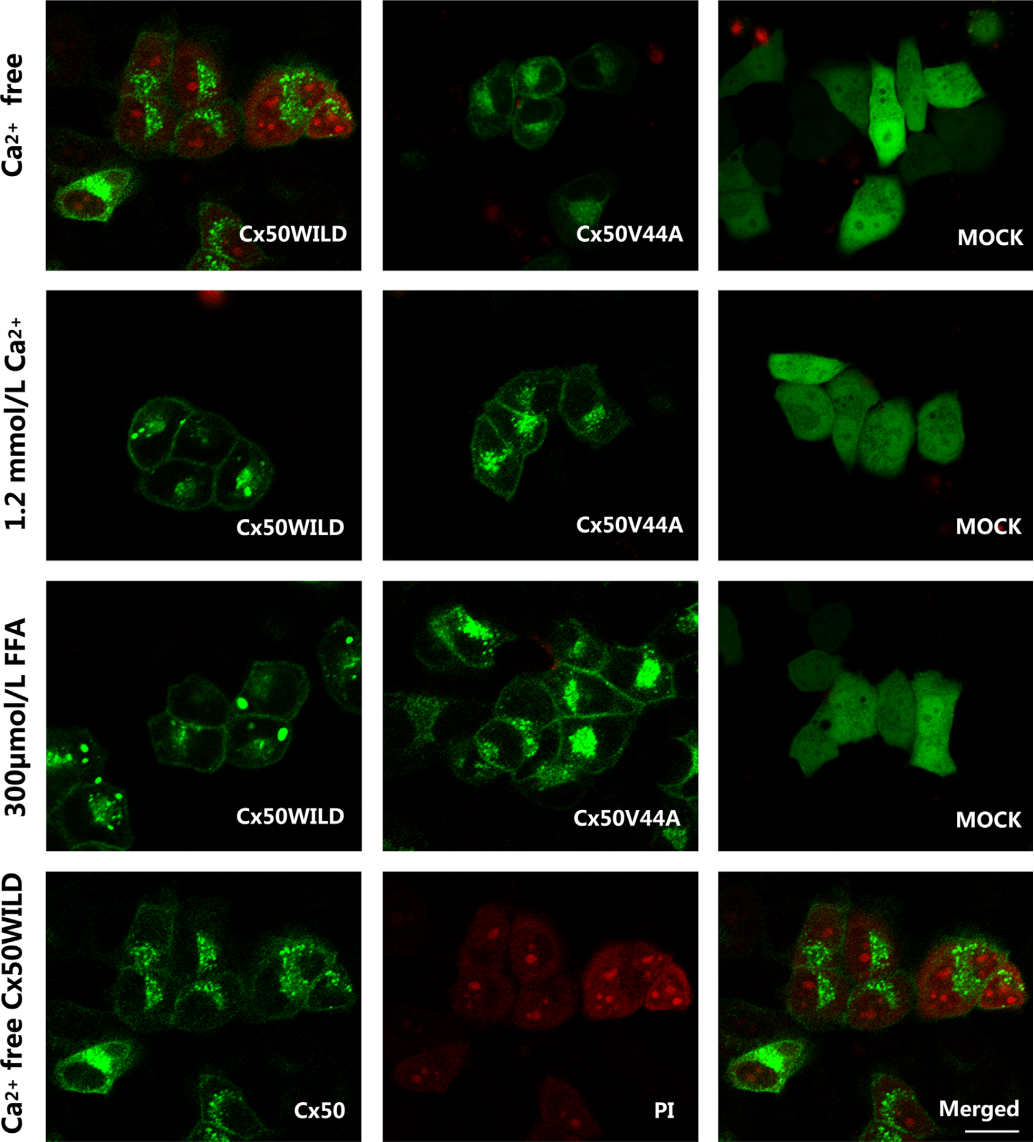
PI dye uptake in HeLa cells stably transfected with Cx50WILD and Cx50V44A. The PI dye uptake assay revealed similar results as the DAPI dye uptake assay. The three pictures in the last line show the Cx50 protein, PI-stained nuclei (nucleoli)/cytoplasm, and merged look of the wild-type cells in Ca^2+^-free HBSS. Scale bar: 20 μm.
